# Complete second branchial cleft fistula in a fifteen-year-old boy: A case report

**DOI:** 10.1016/j.ijscr.2025.110886

**Published:** 2025-01-14

**Authors:** Abdul Basit, Saim Amir, Nukhbat Ullah Awan, Sarmad Javed, Zain Tariq

**Affiliations:** a King Edward Medical University Lahore, Pakistan; b Mayo Hospital Lahore/King Edward Medical University Lahore, Pakistan

**Keywords:** Branchial anomalies, Second branchial arch, Branchial cleft fistula, Congenital neck anomalies, Sinography, Surgical excision

## Abstract

**Introduction and importance:**

The branchial or pharyngeal apparatus, crucial in embryological development, consists of clefts, arches, pouches, and membranes. Anomalies arising from this apparatus particularly involving the second branchial arch, are rare. Among these anomalies, complete second branchial cleft fistulas, with both external and internal openings, are exceptionally uncommon. This case report presents such a rarity in a fifteen-year-old boy, highlighting the clinical presentation, diagnostic approach, surgical management, and outcome.

**Case presentation:**

A fifteen-year-old boy presented with a history of mucoid discharge from an opening on the lateral aspect of the right neck, noticed since birth. Clinical examination revealed a pinhole opening along the anterior border of the sternocleidomastoid muscle. Imaging studies confirmed the diagnosis of a complete second branchial cleft fistula, extending from the right lateral neck to the right tonsillar fossa. Surgical excision using a stepladder approach was performed under general anesthesia, leading to complete resolution of symptoms.

**Clinical discussion:**

Complete second branchial cleft fistula is a rare entity. The diagnosis requires thorough history and examination, imaging, biopsy and surgical excision along with certain period of folllow-up.

**Conclusion:**

Complete second branchial cleft fistulas are exceedingly rare congenital anomalies, typically presenting with mucoid discharge from a neck opening since birth. Diagnosis involves clinical examination and imaging studies, such as sinography with water-soluble contrast. Surgical excision, often via a stepladder approach, remains the mainstay of treatment, resulting in favorable outcomes. Early recognition and prompt intervention are essential for optimal management.

## Highlights

• The report presents a rare case of a complete second branchial cleft fistula in a fifteen-year-old boy.
• There was history of mucoid discharge from a small opening on the right side of his neck since birth.
• Clinical examination and Imaging studies confirmed the presence of a complete second branchial cleft fistula.
• The fistula was surgically excised using a stepladder approach under general anesthesia.
• The patient had an uneventful recovery post-surgery with complete resolution of symptoms.

## 1 Introduction

The Branchial or pharyngeal apparatus consists of clefts, arches, pouches, and membranes. It contributes to the embryological development of the face and neck [1,2]. The pharyngeal clefts or grooves are the lateral-most part and are covered by ectoderm. Only the first cleft contributes to the development of the epithelial lining of external acoustic meatus. The branchial arches lie medial to the clefts and are composed of mesoderm. Each branchial arch has arterial, cartilaginous, muscular, and neuronal components. The first, second, fourth and sixth arches contribute to the development of nerves, bones, ligaments, and cartilage while the fifth arch is rudimentary. The branchial pouches are medial-most and are composed of endoderm. They contribute to the development of thymus, lymph nodes and parathyroid glands etc. [1].

During embryonic development, the second arch grows caudally to envelope the third, fourth and sixth arches and forms a cervical sinus by fusing with the ectoderm caudal to the sixth arch. The ectoderm inside the sinus obliterates and the edges of the sinus fuse. Persistence of parts of second cleft (ectoderm) or second pouch (endoderm) can result in a branchial fistula.

Birth defects of second branchial groove are common, but a complete fistula with an external and internal opening is quite rare [3].

Here, we present a case of a complete second branchial fistula extending from the right lateral neck to the right tonsillar fossa in the oral cavity.

The work has been reported in line with the SCARE criteria [11].

## 2 Case report

A fifteen-year-old boy presented to the ENT outpatient department with the complaint of a mucoid discharge from an opening on the lateral aspect of the right half of the neck. The flow of the discharge had been periodic, with no aggravating or relieving factors, and no associations. The discharge had been present since birth and had been first noticed by the mother. The patient had a previously unremarkable history, with no familial cases. He belonged to a lower-middle class socioeconomic background.

**Fig. 1:**
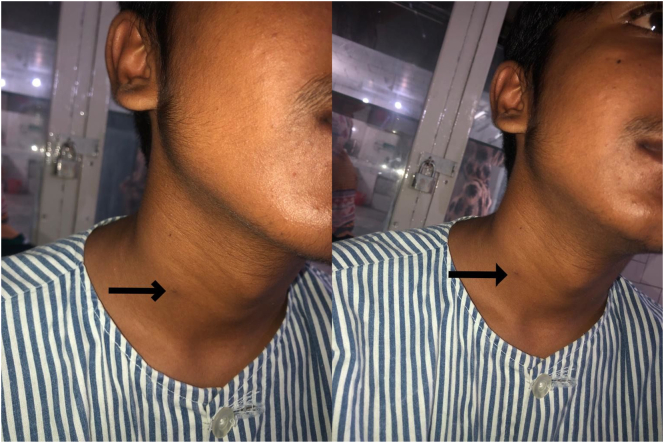
Pre-operative view of the opening of second branchial cleft fistula on the right half of neck as indicated by the arrows.

On examination, the patient was healthy with no visible craniofacial defects. A small, pinhole opening was seen along the anterior border of sternocleidomastoid at the junction of its upper 2/3rd and lower 1/3rd (Fig. 1). At the time of examination, there was no active discharge from the opening. No redness, swelling, tenderness or skin changes were noted in the skin around the opening.

To rule out the possibility of branchio-oto-renal syndrome, kidney profile and ultrasound was done, which revealed normal renal structure and function. Hence, a differential diagnosis of second branchial cleft fistula was made.

**Fig. 2:**
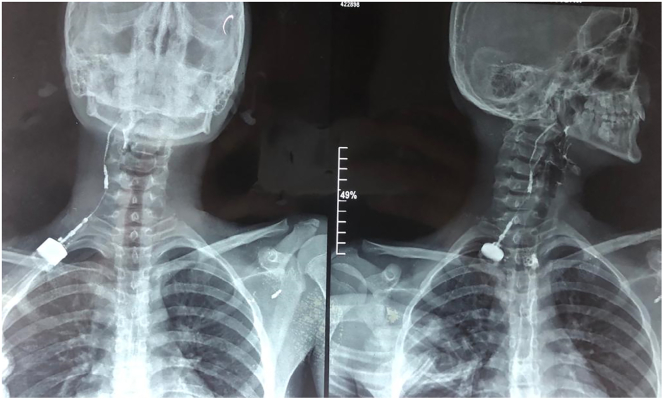
A sinogram with water-soluble contrast revealing a complete second branchial cleft fistula extending from an outer opening on the anterior border of sternocleidomastoid to an inner opening in the tonsillar fossa.

The investigation carried out was a sinogram, with a water-soluble contrast given into the opening with a straight catheter. The contrast was seen outlining a smooth linear tract through an external opening on the right, lateral aspect of neck ascending upwards with the passage of contrast, through the second opening in the posterior part of the right side of the floor of oral cavity (tonsillar fossa) (Fig. 2). This confirmed the presence of a complete second branchial cleft fistula.

**Fig. 3:**
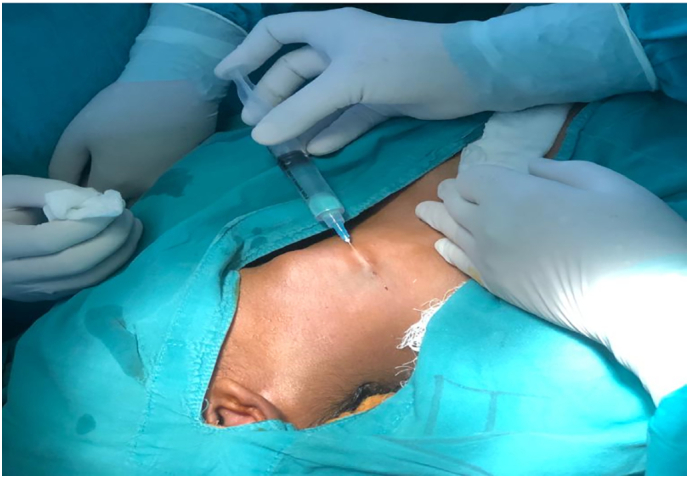
Methylene blue dye being injected to aid the presence of the location of the fistulous tract.

After two weeks, the process of excision of the fistulous tract was done through the stepladder approach, under general anesthesia. The patient was lying supine, with the neck extended. For helping with the identification of the tract, methylene blue dye was instilled (Fig. 3). The tract was found lying between the external and internal carotid arteries, superficial to the hypoglossal nerve.

**Fig. 4:**
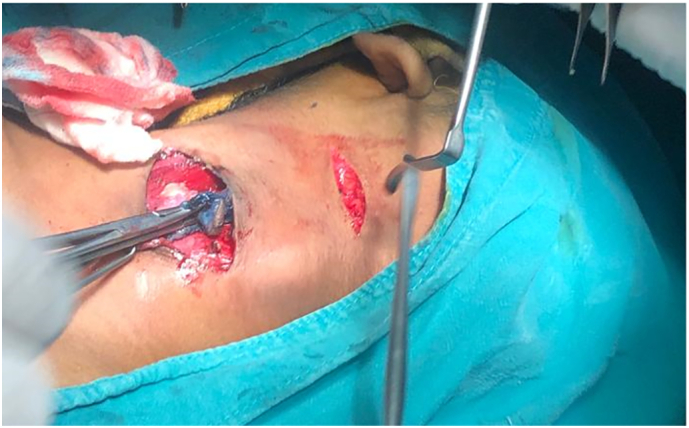
Two incisions given in a stepladder manner for the excision of the tract.

Two transverse elliptical incisions were made in a stepladder fashion (Fig. 4). The first incision was given around the site of the outer opening of the tract, dissected to the subplatysmal plane in depth and then continued superiorly. The second incision was given just above the level of hyoid.

**Fig. 5:**
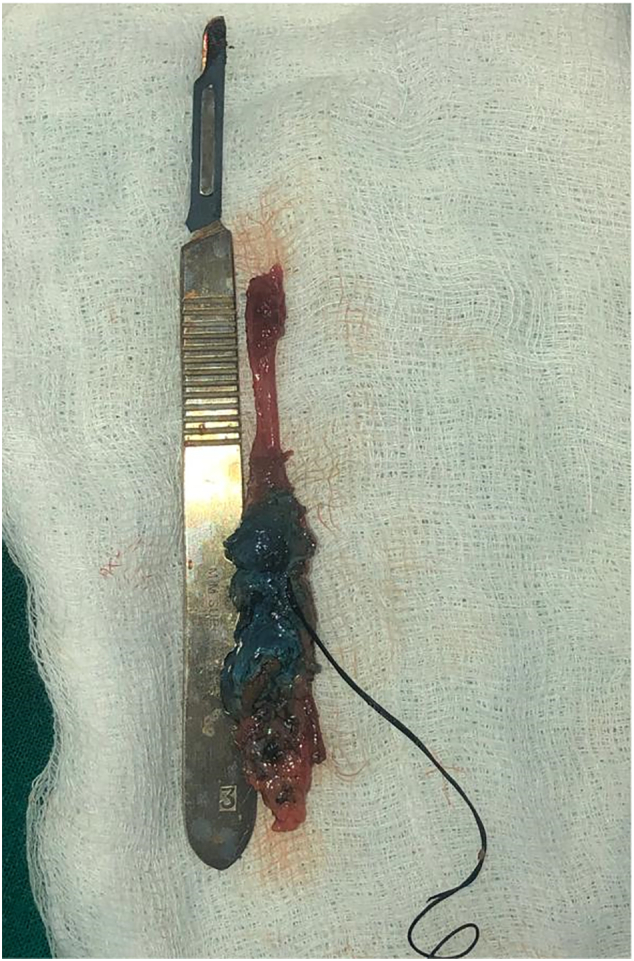
The completely excised fistula placed against a scalpel.

The tract was then completely excised through the upper incision (Fig. 5). The completely excised tract was then sent to histopathology, which confirmed the presence of a right branchial cleft fistula.

The patient was discharged on the fifth post-operative day and was advised to follow-up after a week, and then after a month. The patient showed improvement without any involved complications.

## 3 Discussion

Branchial fistulas are one of the very rare congenital neck anomalies caused by an abnormal development of the pharyngeal apparatus. Branchial anomalies account for 20 % of congenital neck diseases in pediatric population. Anomalies of second branchial arch are relatively common as compared to other branchial arch anomalies and account for 95 % of total branchial arch anomalies [4]. Very few cases are reported for second branchial fistula in Pakistan. However, a complete second branchial fistula is a very rare presentation [5].

These branchial anomalies include branchial cysts, branchial sinus and very rarely a branchial fistula which may be incomplete or complete. A complete second arch branchial fistula is formed by a tract extending between anterior border of sternocleidomastoid muscle and tonsillar fossa and it lies medial to the hyoid bone, lateral to the hypoglossal nerve and makes a passage between external and internal carotid arteries [6].

Bailey classified second branchial arch anomalies into 4 different categories. Type 1 being most superficial, lying under the superficial cervical fascia and located anterior to the sternocleidomastoid muscle. The most frequent type is Type 2, located under the middle cervical aponeurosis and anterolateral to the carotid vessels. And the type which extends to the pharynx is type 3, located between internal and external carotid artery. A very rare type is type 4, which is located lateral and medial to the pharyngeal wall and the carotid artery respectively [7].

A chain of events is required to diagnose a case of second branchial fistula. Clinical history is the first and foremost step in the diagnosis of complete or incomplete second branchial fistula. Clinical history is followed by sinography orfistulography, as done in our case. To outline the structures underlying the tract and extent of the tract, CT angiogram or MRI of the neck region is done [8].

The mainstay of treatment for second arch branchial fistula is surgical excision followed by a course of antibiotics in case of acute infection. Several surgical techniques have been advised for the management of second arch branchial fistula. These techniques involve either using a stepladder approach with two incisions or via a single incision on the anterior border of sternocleidomastoid muscle or a combined pull through technique, with the stepladder approach being the standard procedure. The technique followed in the management of our case was the stepladder approach [9]. Other management options except surgical excision include laser coagulation, chemical cauterization or electrocauterization. These are appreciable approaches in case of neurovascular adhesions or repeated bouts of infection [10].

A complete second branchial arch fistula is a very rare congenital neck anomaly which is diagnosed by clinical history, sinography/fistulograhy, CT angiogram or MRI of the head and neck region. The mainstay of treatment is surgical excision by the gold standard stepladder approach.

## 4 Conclusion

A complete second branchial arch fistula is a very rare congenital neck anomaly which is diagnosed by clinical history, sinography/fistulograhy, CT angiogram or MRI of the head and neck region. The mainstay of treatment is surgical excision by the gold standard stepladder approach. Birth defects of second branchial groove are common, but a complete second branchial fistula with an external and internal opening is quite rare.

## Author contribution

Study conception; Nukhbat Ullah Awan, Saim Amir.

Write up; Abdul Basit, Sarmad Javed, Zain Tariq.

## Consent

Written informed consent was obtained from the patient's parents/legal guardian for publication and any accompanying images. A copy of the written consent is available for review by the Editor-in-Chief of this journal on request.

## Ethical approval

Ethical Approval for Case Reports is not required by the IRB Committee for Ethical Approval-King Edward Medical University Lahore, Pakistan.

## Institutional review board or ethical committee approval

N/A.

## Guarantor

Abdul Basit.

## Research registration number

Not applicable.

## Funding

N/A.

## Conflict of interest statement

There is no conflict of interest among authors.
